# Platelet activation suppresses HIV-1 infection of T cells

**DOI:** 10.1186/1742-4690-10-48

**Published:** 2013-05-01

**Authors:** Theodros Solomon Tsegaye, Kerstin Gnirß, Niels Rahe-Meyer, Miriam Kiene, Annika Krämer-Kühl, Georg Behrens, Jan Münch, Stefan Pöhlmann

**Affiliations:** 1 Institute of Virology, Hannover Medical School, Hannover, Germany; 2 Infection Biology Unit, German Primate Center, Göttingen, Germany; 3 Department of Anesthesiology and Intensive Care Unit, Hannover Medical School, Hannover, Germany; 4 Department of Clinical Immunology and Rheumatology, Hannover Medical School, Hannover, Germany; 5 Institute of Molecular Virology, University Hospital Ulm, Ulm, Germany

**Keywords:** HIV-1, CXCL4, Platelet, Entry

## Abstract

**Background:**

Platelets, anucleate cell fragments abundant in human blood, can capture HIV-1 and platelet counts have been associated with viral load and disease progression. However, the impact of platelets on HIV-1 infection of T cells is unclear.

**Results:**

We found that platelets suppress HIV-1 spread in co-cultured T cells in a concentration-dependent manner. Platelets containing granules inhibited HIV-1 spread in T cells more efficiently than degranulated platelets, indicating that the granule content might exert antiviral activity. Indeed, supernatants from activated and thus degranulated platelets suppressed HIV-1 infection. Infection was inhibited at the stage of host cell entry and inhibition was independent of the viral strain or coreceptor tropism. In contrast, blockade of HIV-2 and SIV entry was less efficient. The chemokine CXCL4, a major component of platelet granules, blocked HIV-1 entry and neutralization of CXCL4 in platelet supernatants largely abrogated their anti-HIV-1 activity.

**Conclusions:**

Release of CXCL4 by activated platelets inhibits HIV-1 infection of adjacent T cells at the stage of virus entry. The inhibitory activity of platelet-derived CXCL4 suggests a role of platelets in the defense against infection by HIV-1 and potentially other pathogens.

## Background

HIV-1 and HIV-2 are the causative agents of AIDS, a major global health crisis, which claimed 1,8 million lives in 2008 [1]. The HIV envelope protein (Env) mediates viral entry into target cells, mainly T cells and macrophages. For this, Env interacts with host cell factors, CD4 and a chemokine coreceptor, CXCR4 or CCR5 [2]. However, HIV can attach to cells independent of CD4 and coreceptor and attachment can modulate viral infectivity and susceptibility to neutralizing antibodies [3]. For instance, binding of HIV to the lectin DC-SIGN on immature monocyte-derived dendritic cells can increase viral infectivity for adjacent target cells [4] and might clear Env from bound neutralizing antibodies [5]. Similarly, erythrocytes, which are CD4 negative, have been reported to selectively bind infectious HIV [6]. Thus, interactions of HIV with receptor-negative, non-susceptible cells can alter viral infectivity and recognition by the immune system and might thus modulate viral spread in and between patients.

Platelets are essential for haemostasis, but also contribute to other fundamental biological processes, including inflammation and defence against invading pathogens [7]. For instance, platelets can selectively kill erythrocytes harbouring the malaria parasite plasmodium falciparum and can protect against spread of the parasite [8]. Several disjointed observations suggest that platelets might also play a role in HIV spread. Thus, thrombocytopenia (low platelet counts) is a frequent complication in HIV-1 infection, afflicting 10-50% of the infected individuals [9]. In addition, platelets counts were found to be associated with viral load and disease progression [10], indicating that platelets might modulate viral spread in patients. Finally, platelets were shown to bind to HIV in cell culture and in infected patients [11-13] and viral capture activity was traced to the calcium-dependent (C-type) lectin DC-SIGN, which was detected on the surface of platelets [14,15]. However, it is at present unclear how HIV capture by platelets impacts viral infectivity.

Platelets are activated upon exposure to e.g. components of the vascular connective tissue and activation induces morphological changes and release of effector molecules stored within platelet granules [16], including the recently identified HIV-1 inhibitor CXCL4 [17]. There is evidence that platelets in HIV infected individuals show signs of activation [18,19], but the consequences of activation for platelet function and HIV interactions are unclear. It is also largely unknown how platelets are activated in the context of HIV infection. Certain adenoviruses can activate platelets via a direct interaction [20] and HIV might do the same, but this scenario has not been experimentally tested.

Here, we provide evidence that platelets inhibit host cell entry of HIV-1 by activation-induced release of CXCL4, an abundant component of α-granules in platelets [21,22]. These results suggest that platelets might constitute a so far unappreciated innate barrier against HIV-1 transmission and spread.

## Results

### Generation and characterization of resting and activated platelets

**Figure 1:**
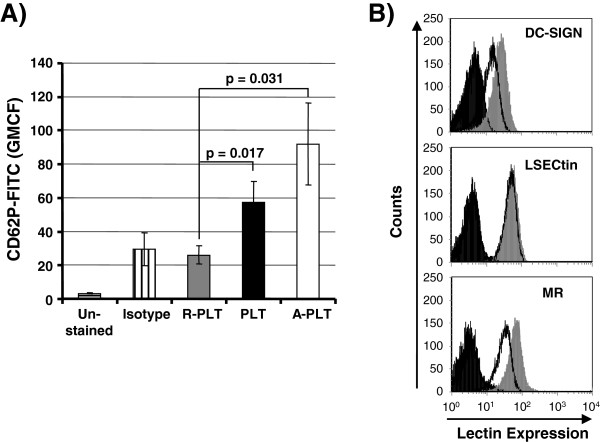
**Analysis of CD62P and C-type lectin expression on platelets.** (**A**) CD62P expression on activated and resting platelets. Platelets maintained resting by treatment with PGE_1_ (R-PLT), activated by exposure to TRAP (A-PLT) or platelets left untreated (PLT) were analyzed by flow cytometry for surface expression of CD62P. The geometric mean channel fluorescence was measured. The results present the average of three experiments with samples from two different donors. Error bars indicate standard deviation (SD). (**B**) Lectin expression on platelets. Untreated platelets were stained with anti-DC-SIGN, -MR and -LSECtin antibodies (grey histograms) or isotype-matched control antibodies (black lines) and analyzed by flow cytometry. Unstained platelets are depicted in black filled histograms. The results are representative of three independent experiments.

We had previously shown that the C-type lectin DC-SIGN facilitates HIV-1 binding to platelets and that bound viruses are infectious for adjacent T cells [15]. The goal of our present study was to determine if platelets modulate HIV-1 spread in co-cultured T cells. In addition, we sought to reveal if the potential impact of platelets on HIV-1 infection depends on the platelet activation status. In order to obtain differentially activated platelets, we either prevented platelet activation by treatment with prostaglandin E1 (PGE_1_), activated platelets by exposure to thrombin receptor agonist peptide (TRAP) or left platelets untreated. Analysis of the expression of CD62P, a platelet activation marker, confirmed that treatment with PGE_1_ and TRAP was effective. Thus, platelets exposed to TRAP expressed high levels of CD62P (activated platelets, A-PLT) while expression on platelets treated with PGE_1_ was close to background (resting platelets, R-PLT) (Figure 1A). Untreated platelets had an intermediate phenotype (untreated platelets, PLT) (Figure 1A). In addition, we investigated if untreated platelets expressed C-type lectins known to bind to the HIV Env protein. Our results demonstrated expression of low levels of DC-SIGN, in agreement with previous studies [14,15], and of mannose receptor (MR) (Figure 1B), a C-type lectin previously shown to promote HIV-1 capture and *trans*-infection by macrophages [23,24]. In contrast, LSECtin, a C-type lectin which captures recombinant HIV-1 Env but not infectious HIV-1 [25,26], was not detected on platelets (Figure 1B) although we cannot rule out that low levels of lectin expression remained undetected due to limited assay sensitivity. Analysis of C-type lectin expression on activated platelets yielded similar results (data not shown), indicating that activation does not modulate C-type lectin expression.

### Platelets suppress HIV-1 spread in a closed cell culture system

**Figure 2:**
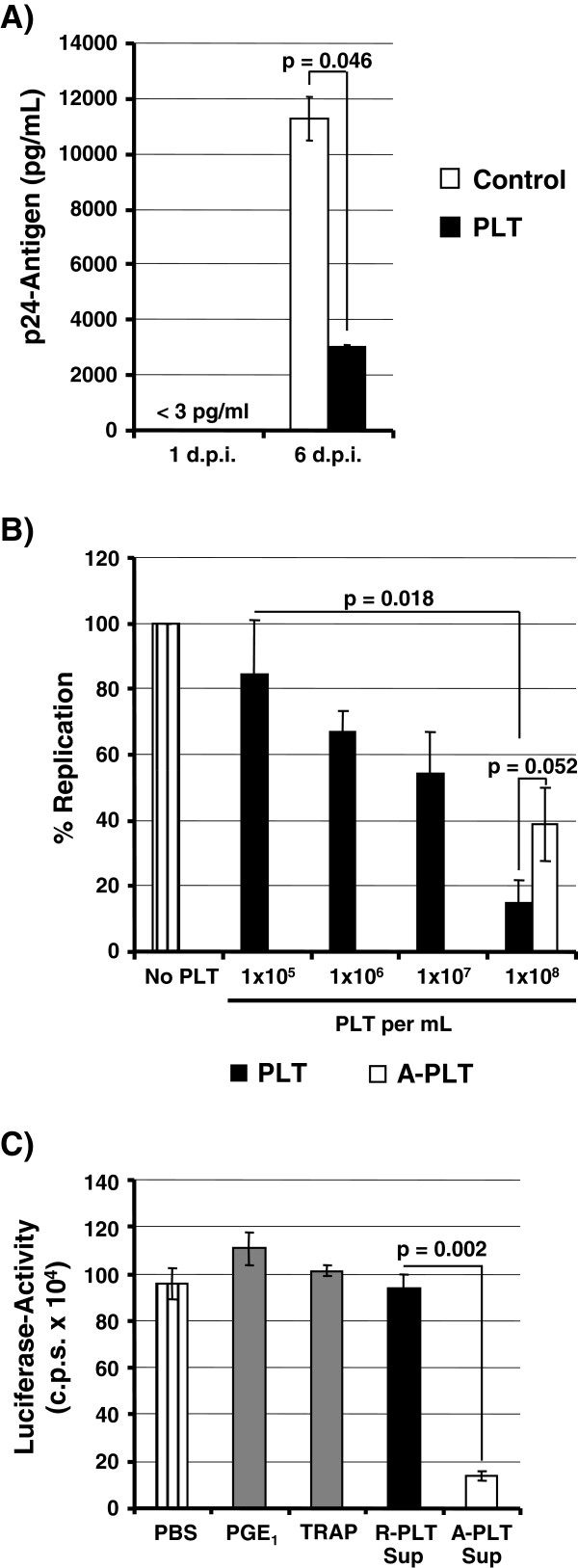
**Platelets inhibit HIV-1 spread in T cells and release an anti-HIV-1 activity upon activation.** (**A**) PHA stimulated PBMCs were infected with 10 pg of HIV-1 NL4-3 in the presence of untreated platelets (PLT, 1 × 10^8^/mL) or medium alone (control) and p24-antigen content in the supernatants was measured on day one and six post infection. The p24-antigen levels at day one post infection were below detection range. The results of a single representative experiment performed in duplicates are shown, error bars indicate SD. The results were confirmed in an independent experiment. (**B**) Platelets inhibit HIV-1 spread in adjacent T cells in a concentration- and activation status- dependent manner. The indicated amounts of non-resting platelets (PLT, platelets left untreated) or activated platelets (A-PLT, platelets treated with TRAP) were added to C8166-SEAP T cells, the cultures infected with 10 pg of HIV-1 NL4-3 and SEAP-activity measured at day five post infection. The average ± SEM of three experiments performed in triplicates is shown, SEAP-activity measured in the absence of platelets was set as 100%. (**C**) Activation of platelets induces the release of one or more HIV-1 inhibitory factors. The indicator cell line TZM-bl was incubated with supernatants from resting (R-PLT Sup) and activated platelets (A-PLT Sup) or incubated with PBS or PBS containing 10 μM PGE_1_ or 100 μM TRAP. Subsequently, the cells were infected with HIV-1 NL4-3 and luciferase activities in the lysates of infected cells were measured. The results ± SD of a single experiment performed in triplicates is shown. Similar results were obtained in a separate experiment.

For the analysis of platelet-mediated HIV-1 *trans*-infection we had previously exposed untreated platelets to a relatively high amount of HIV-1 (ng range of p24 antigen), removed unbound virus after an incubation period and then co-cultivated the platelets with target T cells [15]. While this protocol is suitable to detect HIV-1 capture, it might poorly reflect inter-individual HIV-1 transmission, where generally low amounts of virus are transmitted and cell free virions are present. In order to better mirror the introduction of HIV-1 into the blood stream upon sexual or parenteral transmission, we applied a relatively low amount of virus (pg range of p24 antigen) to co-cultures of platelets and peripheral blood mononuclear cells (PBMCs), omitted a wash step (therefore, the designation closed culture system) and quantified viral spread at six days post infection. Under these conditions, we observed a marked inhibition of HIV-1 spread in the presence of untreated platelets (Figure 2A). To further examine this inhibitory effect, we employed C8166-SEAP T cells which allow convenient quantification of HIV-1 infection [27]. Addition of untreated platelets inhibited HIV-1 spread in C8166 T cells in a concentration-dependent manner, with viral spread being up to 80% reduced in co-cultures harbouring 1 × 10^8^ PLT per mL (a concentration close to that found in human blood) (Figure 2B). HIV-1 inhibition was not due to reduced T cell survival, as determined by annexin V staining (data not shown). Of note, untreated platelets, which largely maintained their granule contents (black bars) were more adept in suppressing HIV-1 than activated and thus degranulated platelets (white bar), which were washed before addition to T cells (Figure 2B). Thus, in a closed culture system platelets can inhibit HIV-1 spread in a concentration- and activation status-dependent manner.

### Platelet activation triggers the release of one or more soluble factors which inhibit HIV-1

The observation that activated and then washed platelets were less able to suppress HIV-1 infection relative to untreated platelets indicated that the content of platelets granules, which is released into the culture supernatant upon activation, might contain one or more factors which inhibit HIV-1. To address this possibility, we incubated TZM-bl indicator cells with supernatants from resting (R-PLT Sup) or activated platelets (A-PLT Sup) prior to infection with replication competent HIV-1 NL4-3. As controls, the cells were treated with PBS or PBS containing TRAP or PGE_1_. The measurement of ATP levels in the cultures showed that neither the substances used to modulate platelet activation nor the platelet supernatants interfered with cell viability (Additional file 1: Figure S1 and data not shown). Pretreatment of the indicator cells with PBS, TRAP, PGE_1_ or supernatants from resting platelets did not impact HIV-1 infection (Figure 2C). In contrast, the supernatants from activated platelets reduced HIV-1 infection by approximately 80% (Figure 2C), demonstrating that factor(s) released upon platelet activation can inhibit HIV-1 infection.

### Supernatants from activated platelets inhibit HIV-1 but not HIV-2 and SIV entry independent of viral strain and coreceptor tropism

**Figure 3:**
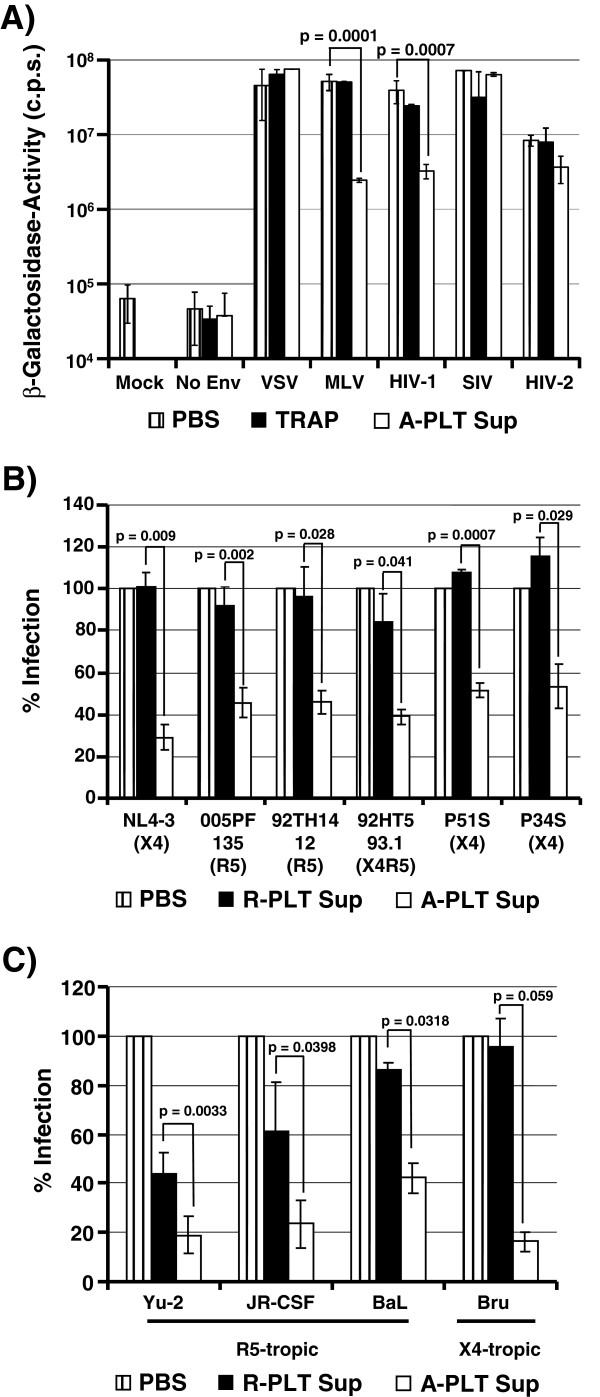
**Supernatants from activated platelets inhibit HIV-1 but not HIV-2 and SIV entry, independent of viral strain and coreceptor tropism.** (**A**) Supernatants from activated platelets efficiently inhibit cellular entry of HIV-1 but not HIV-2 and SIV. TZM-bl indicator cells were preincubated with the supernatants from TRAP-activated platelets (A-PLT Sup) or TRAP containing PBS (TRAP) or PBS. Subsequently, the cells were infected with pseudotypes bearing the indicated Env proteins or with replication competent HIV-2 Rod and SIVmac 239 and β-galactosidase activity in cell lysates was measured. The results ± SD of a representative experiment performed in triplicates are shown. Similar results were obtained in a separate experiment. (**B**) Inhibition of HIV-1 entry by supernatants from activated platelets is independent of the viral coreceptor tropism. The experiment was performed as described for (**A**) but HIV-1 NL4-3 chimeras harboring the V3 loops of the indicated primary HIV-1 isolates were used for infection of TZM-bl cells and luciferase activities in cell lysates were quantified. The results of a single representative experiment performed in triplicates are shown; error bars indicate SD. Similar results were obtained in a separate experiment. (**C**) Different molecular clones of HIV-1 are inhibited by the supernatants from activated platelets. The experiment was performed as in (**B**), but the indicated HIV-1 infectious clones were used. The average of two to four independent experiments performed in triplicates is shown, error bars indicate SEM.

We next assessed if supernatants from activated platelets inhibit HIV-1 infection at the stage of host cell entry and are also active against HIV-2 and SIV. For this, we determined if the supernatants inhibit infection by an envelope-defective lentiviral vector pseudotyped with the Env proteins from HIV-1 NL4-3 or the amphotropic murine leukemia virus (MLV) as well as the G-protein of vesicular stomatitis virus (VSV-G). In addition, we examined inhibition of authentic HIV-2 Rod and SIVmac239. None of the viruses tested were inhibited by TRAP (Figure 3A). The supernatants from activated platelets blocked infection by pseudotypes bearing HIV-1 NL4-3 Env but not VSV-G, although in some experiments a minor decrease of VSV-G-mediated entry was observed, indicating that inhibition of HIV-1 infection occurs at the stage of virus entry. In contrast, infection by HIV-2 Rod and SIVmac239 was not efficiently inhibited and similar results were obtained for SIVmac251 and a macrophage tropic SIVmac239 variant, SIVmac239/316 Env (Figure 3A and Additional file 2: Figure S2). Thus, among primate lentiviruses, the inhibitory activity seems to be largely specific for HIV-1 Env. Finally, entry driven by the MLV Env protein was also inhibited (Figure 3A and Additional file 2: Figure S2), suggesting that HIV-1 and MLV Env might share a binding surface for a platelet-derived inhibitory factor. Alternatively, these proteins might be targeted by at least two separate antiviral activities released from platelets.

We next tested the possibility that the antiviral activity released by platelets might only target HIV-1 with a certain coreceptor tropism or might be specific for certain HIV-1 strains. However, the analysis of a panel of HIV-1 NL4-3 V3 loop mutants, which either use CCR5 or CXCR4 as coreceptor for infectious entry (Figure 3B), and of several HIV-1 molecular clones (Figure 3C) demonstrated that HIV-1 inhibition by platelet supernatants was neither coreceptor nor strain specific.

### CXLC4 in platelet supernatants inhibits HIV-1 infection

**Figure 4:**
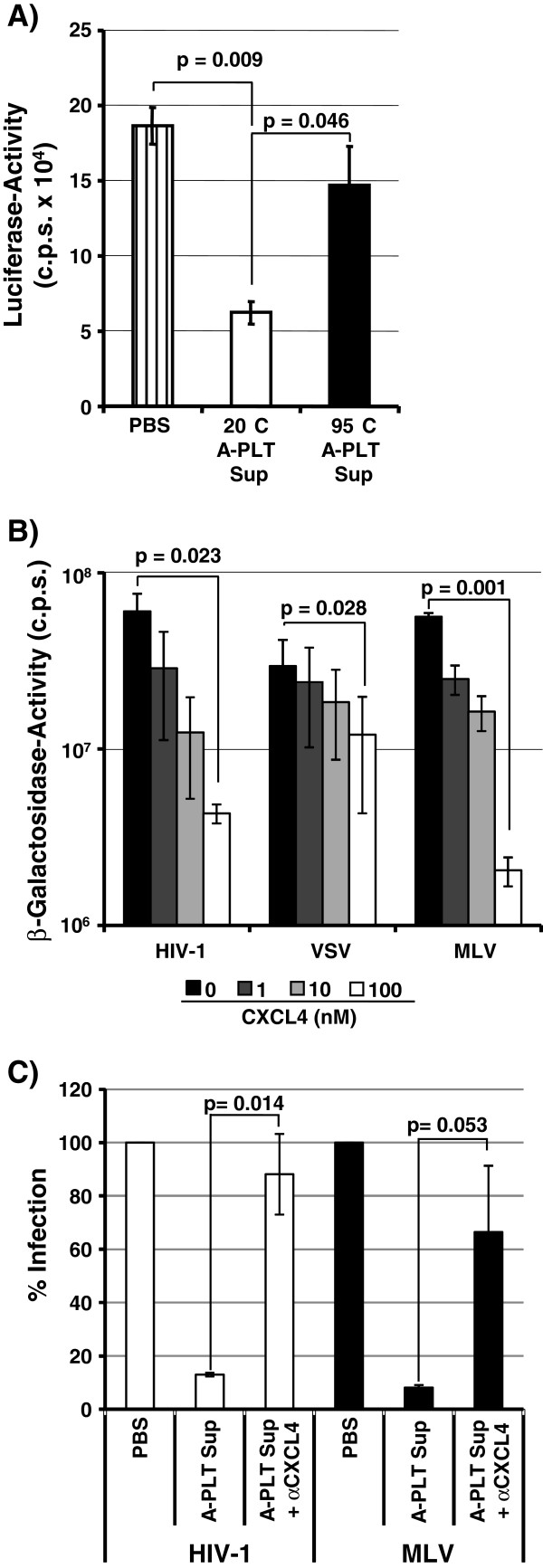
**CXCL4 in supernatants from activated platelets inhibits HIV-1.** (**A**) The anti-HIV-1 factor(s) released from activated platelets are heat labile. Supernatants from activated platelets were left at RT or treated at 95°C for 15 minutes and used to inhibit infection of TZM-bl cells by HIV-1 NL4-3. The results of a representative experiment performed in triplicates are shown and were confirmed in 5 independent experiments, error bars indicate SD. (**B**) Recombinant CXCL4 inhibits HIV-1- and MLV-Env-driven host cell entry. Pseudotypes bearing the indicated Env proteins and normalized for comparable infectivity were used for infection of TZM-bl indicator cells in the presence and absence of recombinant CXCL4. β-galactosidase activities in cell lysates were quantified at 72 h post infection. The results of a representative experiment performed in triplicates are shown; error bars indicate SD. Comparable results were obtained in two independent experiments. (**C**) CXCL4 accounts for the anti-HIV-1 activity of platelet supernatants. Infectivity normalized pseudotypes were incubated with PBS, platelet supernatants or platelet supernatants preincubated with CXCL4-neutralizing antibody and infection of TZM-bl indicator cells was assessed. The results of a representative experiment are shown and were confirmed in two separate experiments. Infection by PBS treated viruses was set as 100%; error bars indicate SD.

To characterize the inhibitory activity released by platelets in more detail, we first asked if it could be inactivated by high temperature. Indeed, incubation of platelet supernatants at 95°C largely abrogated their antiviral activity while supernatants maintained at room temperature were active (Figure 4A). These results are compatible with a proteinaceous factor being responsible for HIV-1 inhibition. While we further characterized this factor, Auerbach and colleagues reported that the chemokine CXCL4, which is abundant in α granules of platelets, inhibits HIV-1 but not HIV-2 and SIV entry [17]. However, these results were exclusively obtained with purified CXCL4 and platelets were not analysed. We therefore investigated if CXLC4 might be responsible for the efficient inhibition of HIV-1 and MLV Env pseudotypes by supernatants from activated platelets. Recombinant CXCL4 markedly inhibited infection driven by HIV-1 or MLV Env but had only a minor (although statistically significant) effect on VSV-G-dependent entry (Figure 4B). Thus, CXCL4 exhibits an inhibitory profile similar to that of platelet supernatants (Figure 3A). A neutralizing antibody against CXCL4 abrogated HIV-1 inhibition by recombinant CXCL4 (Additional file 3: Figure S3) and was used to investigate the contribution of this chemokine to the antiviral activity of platelet supernatants. Incubation of platelet supernatants with the CXCL4-specific antibody largely abrogated inhibition of HIV-1 and MLV Env-bearing pseudotypes (Figure 4C), indicating that CXCL4 in platelet supernatants mainly accounts for their antiviral activity.

## Discussion

Platelets, anucleated fragments of megakaryocytes, have a central role in primary and secondary haemostasis. It is becoming appreciated, however, that platelets have functions beyond ensuring the integrity of the vasculature, which include a role in defence against invading pathogens [7,28]. We found that activation of platelets induces release of one or more antiviral factors, which suppress HIV-1 infection at the stage of viral entry. Inhibition of several HIV-1 strains was observed and the antiviral activity was independent of coreceptor tropism or target cell type. Recombinant CXCL4 inhibited HIV-1 infection and the HIV-1 inhibition by platelet supernatants was largely rescued by a neutralizing CXCL4-specific antibody, indicating a major contribution of CXCL4 to HIV-1 inhibition by platelets. These results suggest that platelets could constitute a so far unappreciated innate defence against HIV-1 infection.

It is well documented that platelets associate with HIV-1 in cell culture and in infected patients [11-13,29,30]. Our previous results [15] and a study by Boukour and colleagues [14] demonstrated that platelets bind HIV-1 mainly via the C-type lectin DC-SIGN and that bound virions are infectious for adjacent target cells [15]. The C-type lectin-like protein CLEC-2 also contributed to HIV-1 capture by platelets but capture efficiency was reduced compared to DC-SIGN [15,31]. Our present study adds MR to the list of C-type lectin (−like) proteins which could contribute to HIV-1 capture by platelets and potentially megakaryocytes. Although a contribution of MR to HIV-1 interactions with platelets remains to be demonstrated, these findings highlight that platelets express several lectins involved in pathogen recognition and might thus modulate pathogen spread and pathogen specific immune responses.

The presence of platelets in HIV-1 infected T cell cultures reduced viral spread efficiently and in a dose-dependent manner. At first sight, this finding is counterintuitive, considering our previous finding that platelets capture and transfer infectious HIV-1 to T cells via lectins [15]. However, different experimental conditions were chosen to generate these results, and the platelet/T cell co-cultures infected with low amounts of HIV-1 in the absence of a wash step (Figure 2A, B of present manuscript) reflect the physiological situation better than the conditions previously chosen to analyse viral capture (high amount of input virus, removal of unbound virus from platelets [15]). The inhibition of HIV-1 observed in these co-culture experiments was maximal when platelet – T cell ratios were used that were similar to those found in human blood, suggesting that platelet-dependent blockade of HIV-1 might occur in patients.

Platelet granules contain hundreds of bioactive molecules which are released into the extracellular space upon platelet activation [16]. Two findings indicate that platelet granules contain an anti-HIV-1 activity, which is released upon activation, and which is largely responsible for the inhibition of HIV-1 spread in platelet/T cells co-cultures: Activated and subsequently washed (and thus granule-deprived) platelets inhibited HIV-1 spread less efficiently than untreated platelets and supernatants of activated but not resting platelets efficiently suppressed HIV-1 infection. Such a scenario raises two immediate questions. How are platelets activated in HIV-1 infected co-cultures with T cells and what is the nature of the antiviral factor? Concerning the trigger for activation, direct contact between HIV-1 and platelets could be sufficient for activation, as previously demonstrated for certain adenoviruses [20]. However, exposure of platelets to virus-like particles bearing HIV-1 Env did not induce appreciable platelet activation (Additional file 4: Figure S4; Additional file 5: Additional methods), arguing against this hypothesis. Alternatively, activation of platelets might be relatively unspecific and could be induced by contact of platelets with T cells or tissue culture plastic during culture – a scenario that we currently favour (Figure 1A and Additional file 6: Figure S5).

Our initial attempts to identify the antiviral factor(s) released by platelets focussed on ligands for the HIV-1 coreceptors CCR5 and CXCR4, the CC-chemokines CCL3 (MIP-1α), CCL4 (MIP-1β), CCL5 (RANTES) and the CXC-chemokine CXCL12 (SDF1), respectively. These chemokines can block HIV-1 entry and release of CXCL12 was detected upon platelet activation (data not shown), in agreement with a previous study [32]. However, the concentration of CXCL12 was below that previously shown to block cellular entry of X4-tropic HIV-1. In addition, neutralizing antibodies directed against CXCL12 did not rescue HIV-1 inhibition and no evidence for downregulation of CXCR4 by platelet supernatants was obtained (data not shown), indicating that platelet-derived CXCL12 was not involved in blockade of X4-tropic HIV-1. A report published during the preparation of this manuscript showed that the chemokine CXCL4 binds to HIV-1 Env and inhibits HIV-1 entry [17]. In contrast, cellular entry of HIV-2 and SIV was not blocked by CXCL4 [17]. CXCL4 is present at high levels in α-granules of platelets [21,22] and we therefore focussed our further analysis on this chemokine. Platelet supernatants and recombinant CXCL4 exerted antiviral activity against HIV-1 without interfering with CD4 and coreceptor expression (Additional file 7: Figure S6) and neutralization of CXCL4 largely prevented HIV-1 inhibition by platelet supernatants, demonstrating that inhibition of HIV-1 infection by activated platelets is mainly due to release of CXCL4. Recombinant CXCL4 and endogenous CXCL4 in platelet supernatants also inhibited MLV Env-driven host cell entry. Whether the blockade of MLV Env by CXCL4 also involves interactions between these proteins or is due to CXCL4 binding to host cell factors required for MLV Env-dependent entry remains to be determined.

Release of CXCL4 by platelets could impact HIV-1 dissemination between individuals. Thus, transmission of HIV-1 via the sexual and particularly the parenteral route frequently involves (micro-) vascular injury, and the resulting platelet activation and release of CXCL4 might reduce transmission efficiency. Platelet-derived CXCL4 might also modulate viral spread during the chronic phase of the infection: Platelets isolated from the blood of HIV-1 patients were reported by several [18,19] but not all [33] studies to express activation markers. Moreover, a recent analysis demonstrated that platelets from HIV-1 infected individuals have a reduced threshold to activation, and that plasma from HIV-1 patients activates platelets obtained from healthy donors [34]. Thus, the activation status of platelets is increased in the context of HIV-1 infection, potentially due to pro-inflammatory cytokines or invading bacteria [34], which are present at elevated levels in HIV-1 patients [35] and are known to activate platelets [36,37]. As a consequence, platelets might constantly release CXCL4, which would explain why viral load and platelet counts were found to be inversely correlated in infected humans [10] and why a direct correlation between CD62P levels and viral load was observed in a recent study [19]. However, it also needs to be noted that CXCL4 can increase HIV-1 replication in macrophages after successful viral entry into these cells [38], suggesting that CXCL4 might impact viral spread via more than one mechanism.

Collectively, platelets can negatively regulate HIV-1 spread in an activation status-dependent manner by release of CXCL4 and might form an innate defence against HIV-1. These findings are in line with the observations that platelets express immune cell lectins and toll-like receptors [39] and employ these receptors to respond to pathogen invasion [39]. Ultimately, proof for a protective role of platelets in HIV-1 infection must come from animal models or patients with specific defects in platelet function.

## Conclusions

The presence of platelets in T cell cultures inhibits HIV-1 spread in an activation status-dependent manner and without eliciting cytotoxic effects. Inhibition occurs at the stage of virus entry into host cells and is observed with HIV-1 but not HIV-2 and SIV. Blockade of HIV-1 entry is due to release of the chemokine CXCL4 by activated platelets. In addition to HIV-1, CXCL4 inhibits cellular entry of vectors bearing the MLV glycoprotein. The antiviral activity of platelet-derived CXCL4 suggests that platelets might play a role in the defense against infection by HIV-1, MLV and potentially other pathogens.

## Methods

### Cell culture

Human embryonic kidney 293T cells were cultured in Dulbecco’s Modified Eagle’s Medium (DMEM). C8166-SEAP and TZM-bl express secreted alkaline phosphatase (SEAP) and luciferase/β-galactosidase, respectively, under the control of the HIV promoter, and were cultured as described previously [27,40]. Peripheral blood mononuclear cells (PBMCs) were isolated from whole blood by Ficoll (Biochrom) gradient centrifugation and stimulated for three days in RPMI 1640 medium supplemented with 5 μg/mL PHA (Sigma) and 20 U/mL IL-2 (Roche). Thereafter, cells were washed, resuspended in RPMI 1640 medium supplemented with 20 U/mL IL-2 (Roche) and seeded at a density of 1 × 10^6^/mL in 96 well plates for infection with HIV-1. All cell culture media contained 100 U/mL penicillin, 100 μg/mL streptomycin and 10% fetal calf serum (PAA laboratories), and all cell cultures were maintained at 37°C and 5% CO_2_ incubator.

### Antibodies and recombinant proteins

Anti-CD206-PE-Cy5, anti-mouse IgG1-PE-Cy5 and anti-mouse IgG1-FITC antibodies were from BD Biosciences. Anti-CD62P-FITC, anti-CD62P-PE, anti-mouse IgG1-PE and anti-DC-SIGN-FITC were from BioLegend. Anti LSECtin antibody was described elsewhere [26]. For neutralization of CXCL4 a neutralizing polyclonal antibody from Axxora was used. Recombinant human CXCL4 was purchased from R&D Systems.

### Platelet isolation, *in vitro* activation and flow cytometric analysis

Blood collection for the study was approved by the local ethic commission (Ethikkommission der Medizinischen Hochschule Hannover, Votum No.3150) and all study participants gave informed written consent for their participation. Venous blood was collected in Monovette 3.8% Citrate Coagulation tubes (Sarstedt). The first 4 mL of blood were discarded to avoid isolation of platelets activated due to the needle stick injury. Platelet rich plasma (PRP) was isolated from the remaining blood within 10 min of blood collection by low speed centrifugation (200 × g) for 7 min. Platelets were then isolated by adding an excess of PBS to the PRP followed by centrifugation at 1500 × g for 10 min. 10 μM prostaglandin E1 (PGE_1_) (Calbiochem) was used to maintain platelets in a resting state (R-PLT). Platelets were activated (A-PLT) by 100 μM of thrombin receptor agonist peptide (TRAP) SFLLRNP, (Ana Spec) for 25 min at room temperature and subsequently washed with PBS. Where indicated, platelets left untreated (designated as PLT) were used for experiments, to avoid interference of PGE_1_ with HIV-1 infection of C8166 cells. The European consensus protocol for the flow cytometric characterization of platelet function [41] was employed for analysis of platelets by flow cytometry. Briefly, 5 × 10^6^ washed platelets were incubated with 10 μg/mL antibody for 15 min at room temperature. Samples were then washed in 20-fold excess of PBS and analyzed immediately by flow cytometry employing a Cytomics FC500 (Beckmann-Coulter) machine. Light scatter and fluorescence signals were acquired using logarithmic amplification and platelets were identified by their characteristic size. Gating was confirmed by platelet specific monoclonal fluorochrome-conjugated antibodies. Generally, 10,000 events were collected per gate and data were analyzed with FCS Express software (De Novo Software).

### Plasmids

Plasmids encoding replication-competent HIV-1 NL4-3 [42], HIV-2 Rod [43], SIVmac239 [44], env-defective NL4-3 [45,46], the G-protein of vesicular stomatitis virus (VSV-G) [47], the envelope proteins (Env) of amphotropic murine leukemia virus [47] and NL4-3 variants bearing heterologous V3-loops have been described previously [48]. An expression plasmid for NL4-3 Env was generated by cloning the NL4-3 env gene into plasmid pCG-IRES-GFP [49] using the MluI and XbaI restriction sites. The identity of the sequence was verified by automated sequence analysis. Molecular clones for HIV-1 BaL, Bru/LAV, JR-CSF and YU-2 were obtained from NIH AIDS Research and Reference Reagent Program.

### Viruses

Production of replication-competent HIV-1 and single cycle reporter virus bearing heterologous glycoproteins has been described previously [42,47]. Briefly, 293T cells were CaPO-transfected with plasmids encoding the proviral DNAs or env-defective proviral DNA in conjunction with an expression plasmid for a heterologous glycoprotein. The culture medium was replaced at 12 h after transfection and supernatants were harvested at 48 h post transfection. Supernatants were passed through 0.45 μm filters, aliquotted and stored at −80°C. Viruses were normalized for equal p24-antigen content by ELISA (SIAC-Fredrick or Advanced BioScience Laboratories) or comparable infectivity, using TZM-bl cells as targets and a commercially available kit (Applied Biosystems) for quantification of β-galactosidase activity.

### Impact of platelets on HIV-1 spread in co-cultured T cells

To analyze the impact of platelets on HIV-1 spread in a closed culture system, washed platelets at a concentration of 1 × 10^8^/mL or 10-fold serially diluted concentrations were co-cultured with 2 × 10^4^ C8166-SEAP T cells or 1 × 10^5^ PBMCs in 96-well plates and infected with equal volumes of cellular supernatants containing HIV-1 NL4-3 at a concentration of 10 pg/well of p24-antigen. Replication in C8166-SEAP cells was measured by quantification of SEAP-activity in culture supernatants at 5 days post infection employing a commercially available kit (Applied Biosystems). For quantification of HIV-1 replication in PBMCs the amount of p24-antigen released in the culture supernatant was quantified at day 1 and day 6 post infection employing an HIV-1 p24-antigen ELISA kit (Advanced BioScience Laboratories).

### Antiviral activity of platelet supernatants

In order to assess the antiviral activity of platelet supernatants, HIV-1, HIV-2 and SIV molecular clones, env-defective HIV-1 NL4-3 pseudotyped with homologous or heterologous glycoproteins or NL4-3 V3-loop variants were normalized for comparable p24-antigen content (250 pg/well) or comparable infectivity and were used to infect TZM-bl cells (seeded at a density of 1 × 10^4^ per well in 96 well plates) in the presence or absence of 30 μL supernatant, obtained from 1 × 10^7^ resting or activated platelets. Medium was changed at 8 h post infection and at 3 days post infection, the cultures were lysed and luciferase or β-galactosidase activities in lysates were determined using commercially available kits (Promega, Applied Biosystems). Heat inactivation of supernatants from activated platelets was performed at 95°C for 15 min.

### Antiviral activity of CXCL4

To determine the antiviral activity of the platelet chemokine CXCL4, TZM-bl indicator cells, seeded in 96-well plates, were preincubated with the indicated concentrations of CXCL4 for 30 min before infection with HIV-1 pseudotypes bearing HIV-1 NL4-3 Env, MLV Env or VSV-G. At 8 h post infection, the medium was replaced by fresh culture medium and the cells incubated for 3 days at 37°C. Thereafter, infectivity was quantified by β-galactosidase assay (Applied Biosystems). In order to determine the contribution of CXCL4 to the antiviral activity of platelets supernatants, the supernatants were incubated with CXCL4 neutralizing antibody at a final concentration of 10 μg/ml. After 15 min incubation at RT, virus was added to the platelet supernatants and the mix was immediately transferred onto TZM-bl indicator cells. Cell culture medium was replaced by fresh medium after 8 h and β-galactosidase activity in cell lysates was measured at 72 h post infection as described above.

### Statistical analysis

Statistical significance was determined using Student’s *t* test for paired samples using Excel software.

## Abbreviations

AIDS: Acquired immunodeficiency syndrome; HIV: Human immunodeficiency virus; DC-SIGN: Dendritic cell-specific intercellular adhesion molecule-3-grabbing non-integrin; CXCL4: CXC chemokine ligand 4.

## Competing interests

The authors declare that they have no competing interests.

## Authors’ contributions

TST and KG conducted most experiments and analyzed data. MK, AK, NRM conducted some experiments. GB provided material and designed experiments. JM performed experiments and analyzed data. SP conceived of the study, designed experiments, analyzed data and wrote the manuscript. All authors read and approved the final manuscript.

## Supplementary Material

Additional file 1: Figure S1 Platelet supernatants do not affect cell viability. TZM-bl indicator cells were seeded in 96-well plates and incubated with the same amount of platelets supernatants (obtained from two donors) as for infection experiments. After 48 h, the ATP levels in the cell cultures were measured employing the CellTiter-Glo assay (Promega) according to the manufacturer’s instructions. The results of a representative experiment performed in triplicates are shown and were confirmed by a second experiment. Error bars indicate standard deviation. R-PLT Sup, supernatants from platelets maintained resting by treatment with PGE_1_; PLT Sup, supernatants from untreated platelets; A-PLT Sup, supernatants from platelets activated by treatment with TRAP. Click here for file

Additional file 2: Figure S2 Supernatants from activated platelets efficiently inhibit HIV-1 but not HIV-2 and SIV entry. The indicated viruses were added to TZM-bl indicator cells in the presence of A-PLT Sup or an equal volume of PBS and infection efficiency was assessed by determining β-galactosidase activity in cell lysates. The average of three to eleven independent experiments is shown, error bars indicate SEM (VSV, MLV, SIVmac251, SIVmac239/316Env = 4, HIV-2 Rod: n = 7; HIV-1 NL4-3, SIVmac239: n = 11). Click here for file

Additional file 3: Figure S3 The anti-CXCL4 antibody does not exert unspecific antiviral effects. TZM-bl indicator cells were preincubated for 30 min with CXCL4 (100 nM) and anti-CXCL4 antibody (10 μg/ml) in the indicated combinations prior to infection with HIV-1 NL4-3. Infection efficiency was assessed by determining β-galactosidase activities in cell lysates. The average of three independent experiments is shown; error bars indicate SEM. Infection measured upon incubation of cells with no inhibitor (PBS) was set as 100%. Click here for file

Additional file 4: Figure S4 HIV-1-like particles do not activate platelets. (A) Whole blood was incubated with the indicated platelet agonists (left column) or Env bearing VLPs (Gag NL4-3 Env) or bald VLPs (Gag no Env) or Mock treated (right column) and platelet aggregation measured by electrode aggregometry. The area under the curve indicates the maximal platelet activation after a total of 20 minutes. The results of a representative experiment done in duplicates (two curves) are shown and were confirmed in two separate experiments. (B) Incorporation of Gag and Env into VLPs. The VLPs used in a (A) were subjected to Western blot analysis employing sera directed against Env (anti gp120) and Gag (anti p55). Click here for file

Additional file 5 Additional methods. Click here for file

Additional file 6: Figure S5 Platelets are activated during culture, irrespective of the presence of HIV-1. Resting platelets were cultured in the presence of HIV-1 NL4-3 or an equal volume of RPMI control medium. Surface expression of the platelet activation marker CD62P was analyzed by flow cytometry at 30 minutes (white bars) and 72 hours (black bars) after culturing. The results of a representative experiment performed with platelets obtained from two healthy donors are shown. Click here for file

Additional file 7: Figure S6 CXCL4 does not modulate expression of CD4 and coreceptor. TZM-bl cells were incubated with CXCL4 (100 nM) or an equal volume of PBS for 4 h at 37°C followed by analysis of receptor and coreceptor expression by FACS. The average of three independent experiments is shown; error bars indicate SEM. Click here for file
