# Supplementary figures and images for: Platelet activation suppresses HIV-1 infection of T cells
Source: Retrovirology. 2013 May 1;10:48. doi: 10.1186/1742-4690-10-48 (PMC3660175; doi:10.1186/1742-4690-10-48)

**Figure S1**

**Tsegaye et al.**

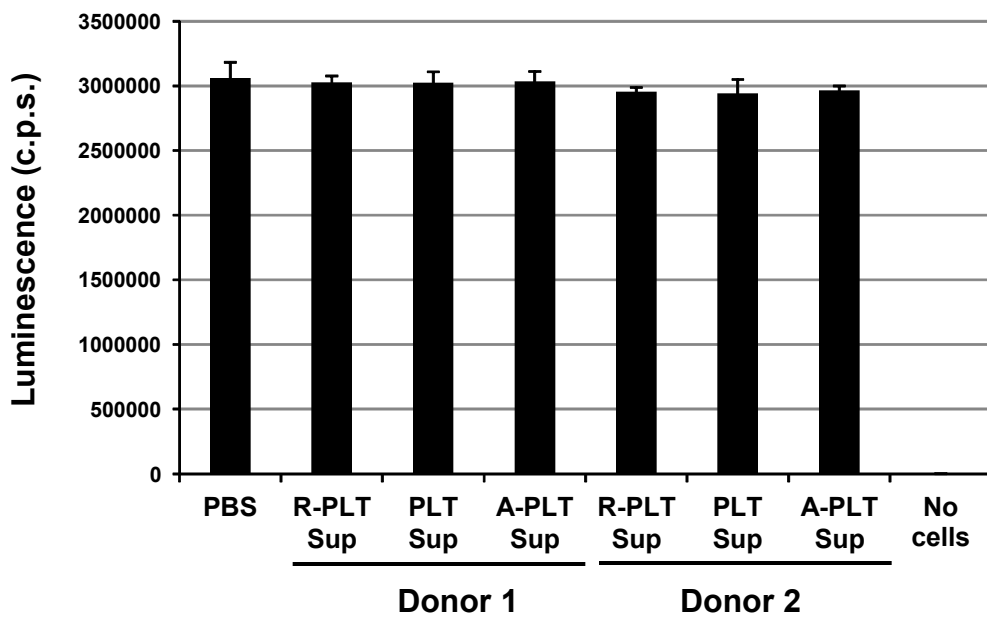

Supplement: Additional file 1: Figure S1 — Platelet supernatants do not affect cell viability. TZM-bl indicator cells were seeded in 96-well plates and incubated with the same amount of platelets supernatants (obtained from two donors) as for infection experiments. After 48 h, the ATP levels in the cell cultures were measured employing the CellTiter-Glo assay (Promega) according to the manufacturer’s instructions. The results of a representative experiment performed in triplicates are shown and were confirmed by a second experiment. Error bars indicate standard deviation. R-PLT Sup, supernatants from platelets maintained resting by treatment with PGE1; PLT Sup, supernatants from untreated platelets; A-PLT Sup, supernatants from platelets activated by treatment with TRAP. [file 1742-4690-10-48-S1.pdf]

Figure S2

Tsegaye *et al.*

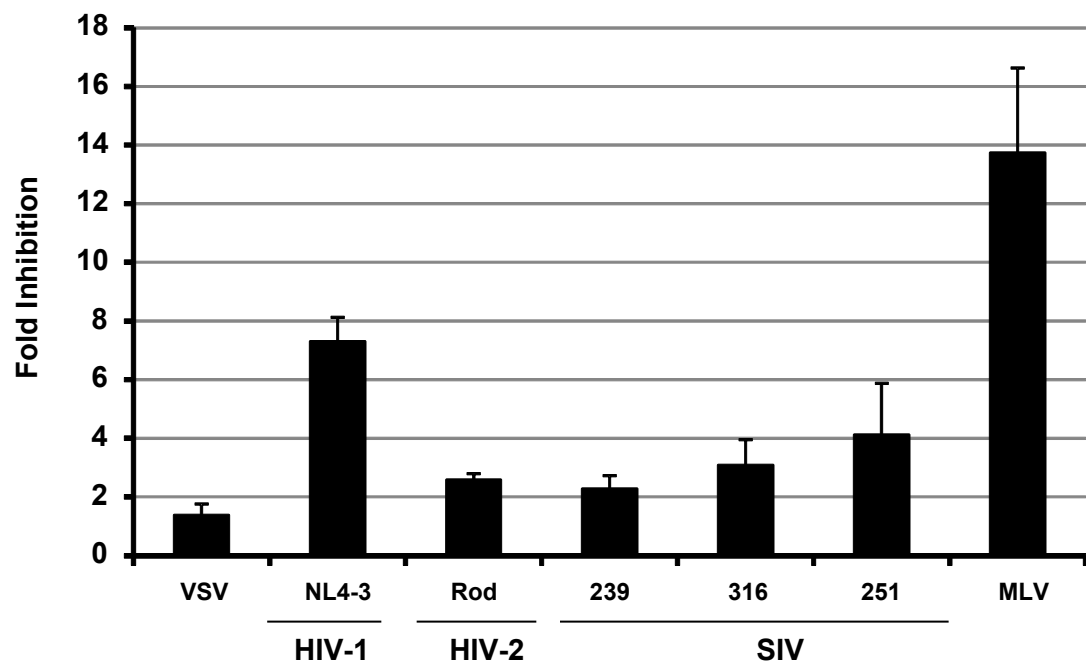

Supplement: Additional file 2: Figure S2 — Supernatants from activated platelets efficiently inhibit HIV-1 but not HIV-2 and SIV entry. The indicated viruses were added to TZM-bl indicator cells in the presence of A-PLT Sup or an equal volume of PBS and infection efficiency was assessed by determining β-galactosidase activity in cell lysates. The average of three to eleven independent experiments is shown, error bars indicate SEM (VSV, MLV, SIVmac251, SIVmac239/316Env = 4, HIV-2 Rod: n = 7; HIV-1 NL4-3, SIVmac239: n = 11). [file 1742-4690-10-48-S2.pdf]

Figure S3

Tsegaye *et al.*

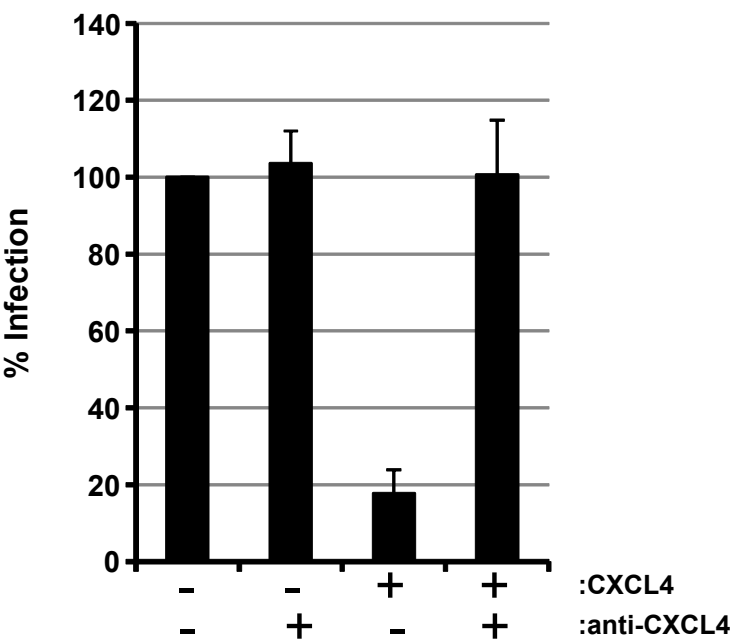

Supplement: Additional file 3: Figure S3 — The anti-CXCL4 antibody does not exert unspecific antiviral effects. TZM-bl indicator cells were preincubated for 30 min with CXCL4 (100 nM) and anti-CXCL4 antibody (10 μg/ml) in the indicated combinations prior to infection with HIV-1 NL4-3. Infection efficiency was assessed by determining β-galactosidase activities in cell lysates. The average of three independent experiments is shown; error bars indicate SEM. Infection measured upon incubation of cells with no inhibitor (PBS) was set as 100%. [file 1742-4690-10-48-S3.pdf]

**Figure S4**

**Tsegaye et al.**

**A)**

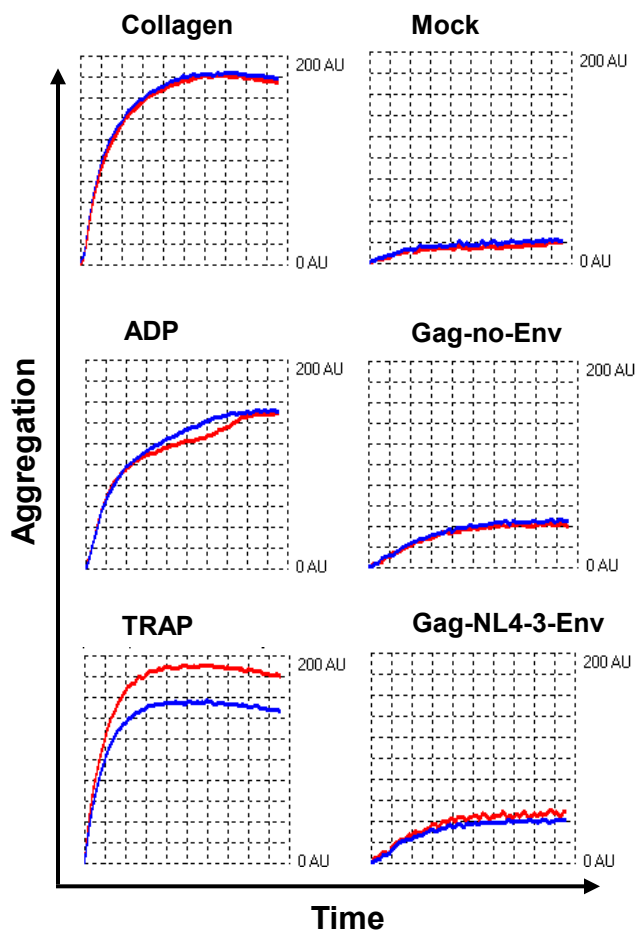

**B)**

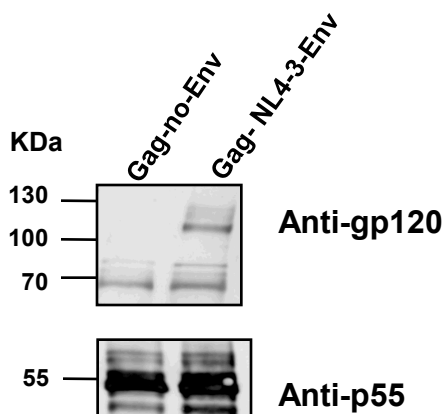

Supplement: Additional file 4: Figure S4 — HIV-1-like particles do not activate platelets. (A) Whole blood was incubated with the indicated platelet agonists (left column) or Env bearing VLPs (Gag NL4-3 Env) or bald VLPs (Gag no Env) or Mock treated (right column) and platelet aggregation measured by electrode aggregometry. The area under the curve indicates the maximal platelet activation after a total of 20 minutes. The results of a representative experiment done in duplicates (two curves) are shown and were confirmed in two separate experiments. (B) Incorporation of Gag and Env into VLPs. The VLPs used in a (A) were subjected to Western blot analysis employing sera directed against Env (anti gp120) and Gag (anti p55). [file 1742-4690-10-48-S4.pdf]

Figure S5

Tsegaye *et al.*

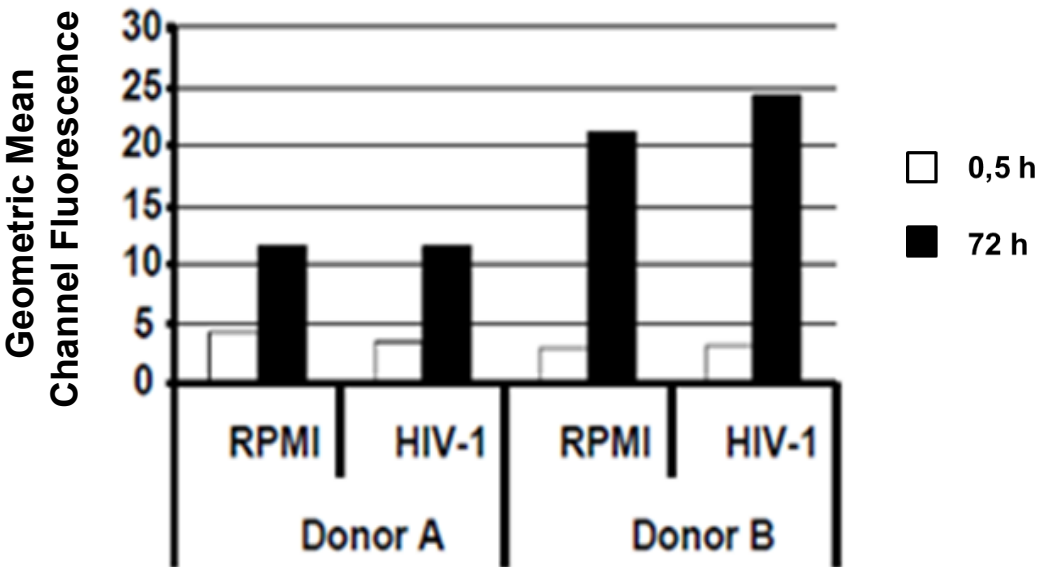

Supplement: Additional file 6: Figure S5 — Platelets are activated during culture, irrespective of the presence of HIV-1. Resting platelets were cultured in the presence of HIV-1 NL4-3 or an equal volume of RPMI control medium. Surface expression of the platelet activation marker CD62P was analyzed by flow cytometry at 30 minutes (white bars) and 72 hours (black bars) after culturing. The results of a representative experiment performed with platelets obtained from two healthy donors are shown. [file 1742-4690-10-48-S6.pdf]

Figure S6

Tsegaye *et al.*

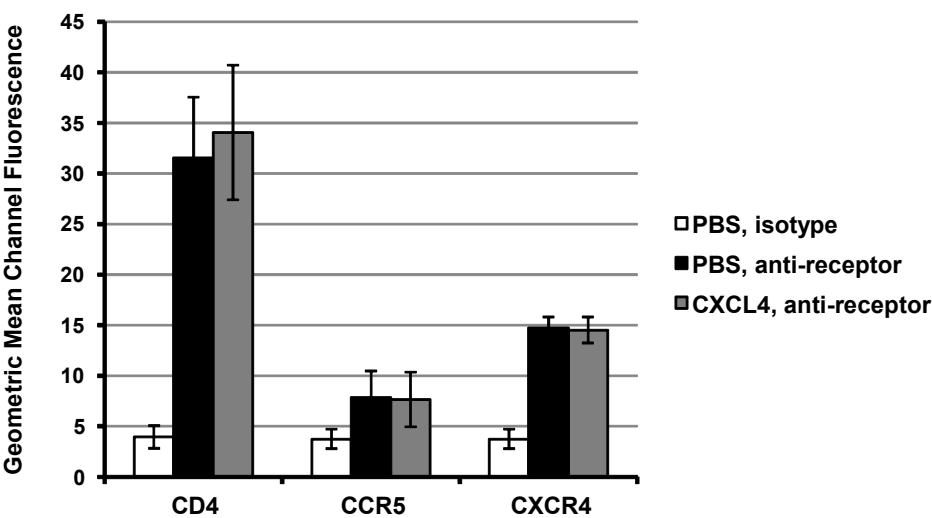

Supplement: Additional file 7: Figure S6 — CXCL4 does not modulate expression of CD4 and coreceptor. TZM-bl cells were incubated with CXCL4 (100 nM) or an equal volume of PBS for 4 h at 37°C followed by analysis of receptor and coreceptor expression by FACS. The average of three independent experiments is shown; error bars indicate SEM. [file 1742-4690-10-48-S7.pdf]
