# Supplementary material for: Platelet activation suppresses HIV-1 infection of T cells
Source: Retrovirology. 2013 May 1;10:48. doi: 10.1186/1742-4690-10-48 (PMC3660175; doi:10.1186/1742-4690-10-48)
Supplement: Additional file 5 — Additional methods. [file 1742-4690-10-48-S5.pdf]

Additional methods

Generation of HIV-1 virus-like particles

For generation of virus-like particles (VLPs), HIV-1 Gag expression plasmid p96ZM651gag-opt (obtained through the NIH AIDS Research and Reference Reagent Program) was co-transfected with plasmid encoding HIV-1 NL4-3 Env or co-transfected with empty plasmid into 293T cells using the calcium phosphate method. The culture medium was replaced by fresh medium at 12 h post transfection and supernatants were harvested at 48 hours post transfection. Supernatants were filtered through a 0.45 µm filter (Sarstedt), concentrated with a Vivaspin centrifugal concentrator (Sartorius) and VLPs were pelleted by ultracentrifugation through a 20% sucrose cushion at a speed of 25,000 rpm for 2 hours at 4°C. Finally, VLPs were resuspended in 100µL 1 x PBS, aliquotted and stored at -80°C until use.

Immunoblot analysis of VLPs

Ten microliters of the indicated VLPs were subjected to SDS-PAGE electrophoresis analyzed by standard Western blot techniques. Mouse anti-gp120 (mab#188) and anti-p24 hybridoma supernatant (183-H12-5C) were used to detect the presence of Env and Gag in VLPs, respectively (detection reagents were from NIH AIDS Research and Reference Reagent Program).

Whole blood impedance platelet aggregometry

Platelet aggregometry was performed on the Multiplate analyser® (Dynabyte medical) to access aggregometric potential of platelets in response to control agonists or VLPs. The measurements were performed as duplicates (red and blue line) at 36.7°C. The impedance change caused by the adhesion of the platelets onto the sensor surfaces is plotted against time (20 min for all samples

tested). The recordings started at the time point when the reagent was added. Control agonist concentrations used were as follows; ADP (ADPtest, 6.5 mM), collagen (COLtest, 3.2 mg/ml) and TRAP-6 (thrombin receptor activating peptide, TRAPtest, 32 mM) that activate the GP IIb/IIIa receptor on the platelet.

Analysis of CD4 and coreceptor expression on CXCL4 treated cells

TZM-bl indicator cells were seeded at 1,4 x 10^5 cells per well 24 h prior FACS analysis. Cells were incubated in culture medium supplement with recombinant CXCL4 at a final concentration of 100 nM or an equal volume of PBS for 4 h at 37°C. After washing with PBS cells were detached with 5 mM EDTA, pelleted and stained with 10 µg/ml mouse anti-CXCR4 (#4083 NIH AIDS Research and Reference Reagent Program), 0,127 µg/ml mouse anti-CD4 (#724) or 25 µg/ml mouse anti-CCR5 (#4088) for 30 min at 4°C. In parallel, cells were stained with isotype-matched control antibodies (obtained from R&D Systems). Subsequently, cells were washed with PBS containing 5 % FCS and 2 mM EDTA and stained with anti-mouse Alexa Fluor 647 (4 µg/ml, Invitrogen) as described above. Staining was analyzed by flow cytometry, employing an LSR II cytometer (BD Bioscience).
